# Lipophilic Statins Eliminate Senescent Endothelial Cells by inducing Anoikis-Related Cell Death

**DOI:** 10.3390/cells12242836

**Published:** 2023-12-14

**Authors:** Barbora Belakova, Nicholas K. Wedige, Ezzat M. Awad, Simon Hess, André Oszwald, Marlene Fellner, Shafaat Y. Khan, Ulrike Resch, Markus Lipovac, Karel Šmejkal, Pavel Uhrin, Johannes M. Breuss

**Affiliations:** 1 Institute of Vascular Biology and Thrombosis Research, Center for Physiology and Pharmacology, Medical University of Vienna, 1090 Vienna, Austriaezzat.awad@meduniwien.ac.at (E.M.A.); andre.oszwald@meduniwien.ac.at (A.O.); shafaatyarkhan@hotmail.com (S.Y.K.); ulrike.resch@meduniwien.ac.at (U.R.);; 2 Institute of Specific Prophylaxis and Tropical Medicine, Center for Pathophysiology, Infectiology and Immunology, Medical University of Vienna, 1090 Vienna, Austria; 3 Department of Pathology, Medical University of Vienna, 1090 Vienna, Austria; 4 Department of Zoology, Government College University Lahore, Lahore 54000, Pakistan; 5 Karl Landsteiner Institute for Cell-Based Therapy in Gynecology, 2100 Korneuburg, Austria; 6 Department of Natural Drugs, Faculty of Pharmacy, Masaryk University, 612 00 Brno, Czech Republic

**Keywords:** endothelial cells, senescence, senolytics, statins, anoikis, apoptosis

## Abstract

Pre-clinical studies from the recent past have indicated that senescent cells can negatively affect health and contribute to premature aging. Targeted eradication of these cells has been shown to improve the health of aged experimental animals, leading to a clinical interest in finding compounds that selectively eliminate senescent cells while sparing non-senescent ones. In our study, we identified a senolytic capacity of statins, which are lipid-lowering drugs prescribed to patients at high risk of cardiovascular events. Using two different models of senescence in human vascular endothelial cells (HUVECs), we found that statins preferentially eliminated senescent cells, while leaving non-senescent cells unharmed. We observed that the senolytic effect of statins could be negated with the co-administration of mevalonic acid and that statins induced cell detachment leading to anoikis-like apoptosis, as evidenced by real-time visualization of caspase-3/7 activation. Our findings suggest that statins possess a senolytic property, possibly also contributing to their described beneficial cardiovascular effects. Further studies are needed to explore the potential of short-term, high-dose statin treatment as a candidate senolytic therapy.

## 1. Introduction

Cellular senescence plays an important role in aging, and studies over the last two decades have shown that accumulating senescent cells can affect many different organs and contribute to numerous age-related pathologies including atherosclerosis, macular degeneration, osteoporosis, neurodegeneration, and sarcopenia [1].

Senescent cells are defined by their permanent loss of the potential to proliferate. Such loss can be induced by stresses, such as DNA damage and telomere dysfunction, increased levels of reactive oxygen species (ROS), or mitochondrial dysfunction [2], and is maintained by modulation of the p16/Rb and p21/p53 axis of cell cycle inhibitors. Senescent cells furthermore develop a pro-inflammatory phenotype, termed the senescence-associated secretory phenotype, SASP [3], in which they secrete pro-inflammatory and matrix-degrading molecules and typically undergo morphological changes such as cell enlargement and flattening [1]. 

Endothelial senescence under physiological conditions is prompted by various factors within the vascular microenvironment as they can be found in atherosclerotic plaques [4]. Disturbed hemodynamic flow, especially in areas like branching arteries and the aortic arch, induces a high rate of cellular turnover and contributes to senescence [5,6,7]. Elevated oxygen levels within arteries present an additional challenge to endothelial cells. Prolonged exposure to factors associated with Western lifestyles, such as high glucose levels, insulin, and reactive oxygen species (ROS), can promote senescence. Imbalances in vasodilators and vasoconstrictors, along with impaired nitric oxide (NO) availability, create an environment conducive to senescence. Chronic inflammation, often associated with increased ROS, exacerbates the process. These factors collectively regulate endothelial cell senescence in physiological contexts, as comprehensively reviewed in [2]. 

Pioneering pre-clinical experimental animal model studies over the past decade have shown that the elimination of such malfunctioning pro-inflammatory senescent cells can ameliorate age pathologies, improve the overall health status, and extend the life span of experimental animals [8,9,10,11,12]. In particular, the removal of senescent endothelial cells could promise broader health benefits [2]. Endothelial senescence increases monolayer permeability [13] and induces expression of leukocyte-attractive (recruiting) adhesion molecules [14]. Senescent endothelial cells also impede the regenerative capacity and functionality of blood vessels, e.g., reduce the availability of the vasodilatory factors nitric oxide (NO) and prostacyclin [15], further supporting the notion that the elimination of senescent endothelial cells could be of clinical importance in vascular homeostasis and tissue regeneration and remodeling and thus, fundamental in the fields of angiology and cardiology.

Collectively, these studies support the idea that the administration of “senolytics”, compounds that preferentially promote the elimination of senescent cells over non-senescent ones could become a therapeutic approach to counteract age-related pathologies. 

Statins represent widely used lipid-lowering drugs utilized for the state-of-the-art treatment of patients with high risk of cardiovascular disease. The reported beneficial effects of these 3-hydroxyl-3-methyl-glutaryl-coenzyme A (HMG-CoA) reductase-inhibiting drugs [16] are attributed to their lipid-lowering [17] and anti-inflammatory properties, including the suppression of leukocyte-endothelial interactions [18,19]. Furthermore, statins promote the synthesis of endothelial NO, an important regulator of blood vessel stiffness that mediates vasorelaxation [20]. In addition, though, when applied at higher concentrations, statins have shown a cytotoxic effect [21,22].

In our study, we investigated whether this cytotoxic effect of statins might show a certain selectivity toward senescent versus non-senescent endothelial cells, which would thereby demonstrate a senolytic capacity of this substance class. We addressed this question by quantifying the cytotoxic effect of statins on two different types of senescent human vascular endothelial cells (HUVECs) and comparing it with the effect on non-senescent HUVECs. We also addressed the dependency of such cytotoxic capacity on the blocking of the mevalonate pathway and the kind of cell death that is induced by statins in endothelial cells.

## 2. Materials and Methods

### 2.1. Isolation, Culturing, and Microscopic Characterization of HUVECs

HUVECs were isolated from human umbilical veins based on the granted permission GS4-EK-4/562-2018 issued by the Ethic Commission of Lower Austria, Austria. Upon isolation, using collagenase NB 4 (Serva, Heidelberg, Germany) as previously described [23], the cells were cultured in M199-based medium supplemented with 20% fetal bovine serum (FBS, Sigma-Aldrich, Saint Louis, MO, USA), 0.4% endothelial cell growth supplement with heparin (ECGS/H, PromoCell, Heidelberg, Germany), and 2 mM L-glutamine, 0.1% penicillin, 0.1% streptomycin, and 0.25 μg/mL fungizone (all from Lonza, Visp, Switzerland), designated as complete endothelial growth medium, on cell culture dishes pre-coated with 1% gelatin (Sigma-Aldrich). Primary cells obtained from nine single-donor cords were pooled at passage 4 (P4). They were frozen or further propagated using serial passaging until doubling times of approximately 10 days were reached. Associated limitations, such as possible bias by donor cells reaching replicative senescence earlier than others, are acknowledged and might in part be alleviated by the juxtaposition to the second model of senescence.

For splitting, the complete endothelial growth medium was removed, and the cells were washed once with calcium- and magnesium-free Hanks’ balanced salt solution (CMFH). The cells were briefly exposed twice to a minimal volume of trypsin-EDTA (Lonza) that was immediately removed, avoiding the need for subsequent centrifugation. Throughout the serial passaging, morphological changes in the HUVECs were monitored with imaging using a Nikon TMS inverted phase-contrast microscope (Nikon, Tokyo, Japan). 

To prepare a stress-induced senescent population of HUVECs, cells at P9 (originating from the same pool of donors), were grown to sub-confluency, trypsinized, and exposed in suspension to 10 Gy of ionizing radiation provided by the cesium^137^-based irradiator IBL-437 C (at the Department of Blood Group Serology and Transfusion Medicine, Medical University of Vienna). The irradiated HUVECs were re-seeded to a growth area six times larger than provided before to ensure enough space for their expected increase in size. During the entire subsequent cultivation period lasting for 10 days, the irradiated HUVECs were observed regularly, and morphological changes were recorded using phase-contrast microscopy as described above. In some experiments, HUVECs were continuously monitored (at a rate of 15 frames per hour) using an Olympus IX83 cellVivo live cell imaging system (Olympus, Tokyo, Japan).

### 2.2. Immunocytochemical Characterization of the Proliferation Status of HUVEC Cultures

At passaging, cell numbers of trypsinized HUVECs were determined by counting with the hemocytometer Luna-II (Logos Biosystems, Anyang-si, Republic of Korea), and their proliferation status was characterized immunocytochemically, assessing the expression levels of the proliferation marker Ki-67 one or two days after passaging, using a rabbit monoclonal anti-Ki-67 antibody (RM-9106-S1, Thermo Fisher Scientific, Waltham, MA, USA). In addition to the assessment of Ki-67, antibodies against several additional targets were used for the characterization of the proliferation status/competence of HUVECs. These included cyclin-dependent kinase inhibitor p16 (MA5-27905, rabbit monoclonal, Thermo Fisher Scientific), a marker for double-strand breaks γH2AX (613402, mouse monoclonal, BioLegend, San Diego, CA), as well as the anti-apoptotic proteins XIAP (2045, rabbit monoclonal, Cell Signaling Technology, Danvers, MA, USA) and Bcl-2 (AF6139, rabbit polyclonal, Affinity Biosciences, Cincinnati, OH, USA). 

For this, prior to staining, HUVECs were fixed for 10 min in 4% paraformaldehyde (PFA, Sigma-Aldrich), washed with phosphate-buffered saline (PBS), permeabilized with 0.2% Triton-X 100 (Serva), and blocked with 3% goat serum (Abcam, Cambridge, UK). The cells were then incubated with the primary antibodies (diluted in antibody-diluent, Dako, Glostrup, Denmark) overnight at 4 °C. After washing with PBS, the cells were exposed to secondary polyclonal antibodies, including goat anti-rabbit IgG Alexa 647 or goat-anti-mouse IgG Alexa 488 (A21245 and A11029, both from Thermo Fisher Scientific) for 2 h at room temperature. Nuclear counter-staining was performed using Hoechst 33258 (CAY-16756-50, Cayman Chemical, Ann Arbor, MI, USA). Immunofluorescent detection was performed using the Olympus IX83 cellVivo live cell imaging system and 10× or 20× magnification. 

Assessment of the senescence markers Ki-67 and γH2AX from fluorescent images was performed with manual counting. At least five different fields of view (comprising at least 500 cells) were assessed and analyzed with an unpaired *t*-test using GraphPad Prism version 5.04 for Windows (GraphPad Software, Boston, MA, USA). The significance level **** indicates *p* < 0.0001.

Differences in p16 expression were assessed as integrated cellular fluorescent signal intensities per field of view divided by the number of cell nuclei present (given in arbitrary units (a.u.)) by assessing at least five different fields of view using the Olympus software package cellSens 2.1 and analyzed with an unpaired *t-*test using GraphPad Prism. The significance level **** indicates *p* < 0.0001.

### 2.3. Monitoring the Senolytic Effects of Test Compounds on HUVECs with Microscopy-Based Cell Enumeration

The senolytic effects of the test compounds were evaluated on HUVECs cultured in a 384-well plate format. The following substances were examined for their senolytic activity: simvastatin (MCE-HY-17502, MedChem Express, Monmouth Junction, NJ, USA), atorvastatin (S5715, Selleck Chemicals, Houston, TX, USA) and lovastatin and pravastatin (SC-200850A and SC-203218, both from Santa Cruz Biotechnology, Dallas, TX, USA) as well as activated simvastatin. 

Statins were applied to HUVECs either alone or in combination with mevalonic acid (90469, Sigma-Aldrich). All non-activated statins, the positive control substances quercetin (PHR1488, Sigma-Aldrich) and dasatinib (HY-10181, MedChem Express), and mevalonic acid were prepared as 10 mM stocks by directly dissolving in DMSO and diluting to working concentrations by mixing with complete endothelial growth culture medium. Activated simvastatin was prepared using alkaline hydrolysis as previously described [24]. Briefly, 8 mg of simvastatin was dissolved in 200 µL of absolute ethanol, followed by the addition of 300 µL 0.1 N NaOH and incubation for 2 h at 50 °C. Finally, 500 µL of sterile water was added to obtain a 20 mM stock. For the negative control treatment, we used complete endothelial growth medium only since the DMSO in the stocks became diluted at least 1000-fold in complete endothelial growth medium during application.

To evaluate the senolytic effects of the tested compounds, the cell death-inducing effects were examined on two types of senescent HUVECs and compared with the effect on proliferation-competent HUVECs (“young”). The first type of HUVECs was exhaustively propagated to senescence (“old”), and the second type was brought to senescence with γ-irradiation (“irradiated”). The irradiated cells were freshly prepared for each experiment, starting approximately 14 days in advance, while “young” and “old” cells were used directly after recovering from storage in liquid nitrogen. Three independent experiments were conducted, each with duplicates.

For the analysis, HUVECs were trypsinized, and transferred into pre-gelatinized (0.2% gelatin) Corning 384-well black plates (3985, Corning, Corning, NY, USA). The cells were re-suspended in complete endothelial growth medium and seeded at a low seeding density of 750 cells per well for young HUVECs, providing sufficient space for proliferation, and 1500 cells for old and irradiated HUVECs, which were larger and non-proliferative. After 24 h of cultivation, and again after 72 h, the culture medium was partially removed (leaving 20 µL to prevent drying out), and the test substances were added to achieve a 1× working concentration. The HUVECs were then exposed to the test and control substances for a period of four days in a CO_2_ incubator. 

Cell fate and the cell count of HUVECs were monitored daily using the Olympus IX83 cellVivo live cell imaging system. Each day, the 384-well plate was temporarily transferred to a temperature- and CO_2_-controlled incubation chamber (PeCon, Erbach, Germany) for a short visualization of each well. At the end of the experiments, the cells were fixed with 2% PFA, washed three times with PBS, permeabilized with 0.2% Triton-X 100, and stained with Hoechst 33258. The daily cell counts as well as end-point cell counts of stained cells, were determined using cellSens software 2.1 (Olympus) and respective macros.

### 2.4. Western Blotting

Cultures of young, old, and irradiated HUVECs were kept in T25 cell culture flasks and exposed for 72 h to either 0.33 µM, 0.6 µM, or 1 µM activated simvastatin or were left in full growth medium as a negative control. Medium renewal was performed after an initial 48 h. As a positive control for apoptosis induction, young HUVECs were exposed for 3.5 h to 0.2 µM staurosporine (HY-15141, MedChem Express). After the 72-hour cultivation period in the presence or absence of activated simvastatin or 3.5 h exposure to staurosporine, respectively, the proteins from adhering cells were isolated. In addition, proteins were isolated from the culture supernatants containing cellular debris of HUVECs subjected to statin treatment (collected after 48 h during medium change). 

Briefly, adhering cells were harvested using trypsinization and centrifuged at 365× *g*, and the obtained pellet was washed twice with ice-cold PBS and then lysed using 100 μL per 10^6^ cells RIPA buffer (Sigma-Aldrich) supplemented with a protease inhibitor cocktail (Bimake, Houston, TX, USA). The lysates were immediately vortexed, placed on ice for 30 min, and centrifuged at 14,000× *g* at 4 °C for 30 min. The pellet containing the cell debris was discarded, and the supernatant was transferred to a new tube and immediately frozen. In the case of culture supernatants, the collected fluids were ultracentrifuged for 45 min at 4 °C and 100,000× *g* in an Optima TLX Ultracentrifuge using the TLA 100.3 rotor and suited polycarbonate tubes (all Beckman Coulter, Brea, CA, USA). The obtained pellets were resuspended in 40 µM protease inhibitor-supplemented RIPA buffer and processed analogously to protein lysates from adhering cells.

A spectrophotometric assessment of protein concentration was carried out using the NanoDrop 2000 instrument (Thermo Fisher Scientific). About 20–30 µg of total protein was mixed with an equal volume of 2× reducing Laemmli solution and heated at 95 °C for 7 min on a thermomixer. Protein samples as well as a pre-stained protein marker (26619, Thermo Fisher Scientific) were loaded and run on a 10% polyacrylamide gel. After separation, the proteins were transferred to polyvinylidene difluoride (PVDF) membranes, (Carl Roth, Karlsruhe, Germany) pre-soaked in methanol, using wet blotting. The membranes were subsequently blocked overnight in 5% skimmed milk (1% fat content; AppliChem, Darmstadt, Germany) in PBS-T. Rabbit polyclonal β-actin antibody (1:1000; A2066, Sigma-Aldrich), rabbit monoclonal lamin B1 antibody (1:500; ab133741), or Apoptosis Western Blot Cocktail (1:250; ab136812) comprising antibodies against pro/p17-caspase-3, cleaved PARP-1, and actin (both from Abcam), were used as primary antibodies. For β-actin and lamin B1 detection, donkey HRP (horseradish peroxidase)-conjugated secondary anti-rabbit (1:5000; NA934, Amersham, Amersham, UK) was used. For Apoptosis Western Blot Cocktail detection, HRP conjugated secondary antibodies (1:200; goat anti-mouse and anti-rabbit) provided in the kit were applied. The membranes were exposed to HRP Western Bright Sirius substrate (Advansta, San Jose, CA, USA), and the resulting chemiluminescence was recorded using a CCD-based camera (Alpha Innotech, San Leandro, CA, USA). The obtained images were analyzed using ImageJ software (version 1.53c) [25] and OlyVIA software (version 2.9, Olympus).

### 2.5. Live Cell Imaging and Real-Time Detection of Apoptosis 

Replicative senescent HUVECs were exposed to 0.33 µM activated simvastatin. On day two of exposure, IncuCyte^®^ Caspase-3/7 Green Apoptosis Assay Reagent 4440 (Essen Bioscience, Ann Arbor, MI, USA), enabling real-time visualization of caspase-3/7 activation in cells, was added to the complete endothelial growth medium. Time-lapse sequences were recorded over 15 h (1 frame per 6 min) with the Olympus IX83 cellVivo live cell imaging system using both the appropriate fluorescent channel as well as bright-field imaging.

### 2.6. Statistical Analysis

Daily recorded images of the 384-well test plates of three independent experiments performed in duplicates were analyzed using the Olympus cellSens software’s Count and Measure module. In preparation for parametric statistical testing, Shapiro–Wilk tests were performed on all tested groups to confirm the assumption of a normal distribution, which was confirmed for 77 of 78 conditions. A two-way analysis of variance (two-way ANOVA) showed significant effects of both substance and concentration applied on all three tested cell types. Finally, in order to ascertain which treatment groups differed significantly from their respective controls, a Dunnett’s Multiple Comparison test was performed post hoc. All statistical tests were calculated in R using the packages: “tidyverse” for data processing and plotting [26] and “DescTools” for statistical tests [27].

Concentration–response models were fit from the final-day responses of five substances: simvastatin (both, naïve and activated), lovastatin, atorvastatin, and pravastatin, for all three types of HUVECs. All curves were modeled and plotted using R with the additional packages “drc” [28] and “ggbreaks” [29]. All models were calculated using log–logistic regression functions, with either one or two fixed parameters [30]. The general 4-parameter log–logistic function is described with the following equation:

$$f\left(x\right)=c+\frac{d-c}{1+exp(b\left(log\left(x\right)-log\left(e\right)\right)}$$

For most cases, both lower and upper asymptote limits were fixed (parameters *c* and *d*, respectively), while only the parameters for the slope and the point of inflection (parameters *b* and *e*, where *e* corresponds to the approximate relative ED50) were estimated using functions provided in the “drc” package.

Furthermore, all mean functions of the concentration–response curves were assessed for their fit to their respective data. Lack-of-fit tests showed that the mean structures of the models accurately represent the given data.

Significance was assigned to *p* values less than 0.05. Results are given as means ± SD.

## 3. Results

### 3.1. Generation of the Cells for the Two Types of Senescence Models, of Replicative Senescent HUVECs with Extensive Passaging, and of Stress-Induced Senescent HUVECs with Exposure to γ-Irradiation 

We studied the senolytic aptitude of the tested compounds in two different senescence models. On the one hand, we established replicative senescent cells by extensively propagating a pool of proliferation-competent HUVECs, and on the other hand, we created stress-induced senescent cells by exposing young HUVECs to 10 Gy of ionizing irradiation (“irradiated” HUVECs).

For the induction of replicative senescence, we kept splitting HUVEC cultures and monitored their proliferation capacity and morphology during the whole cultivation period. With an increasing number of passages, the endothelial cultures became less proliferative, and the cells gradually changed their morphological appearance. Specifically, the cells progressively lost their typical cobblestone-like morphology (a hallmark of confluent young HUVECs), became larger, more flat, and frequently multinucleated, and formed long and branched protrusions (Supplementary Figure S1a). To generate cells that became senescent prematurely, with stress exposure, we exposed HUVECs to ionizing radiation. A single dose of 10 Gy induced cell death in approximately 30% of cells. The surviving portion of irradiated cells exhibited features of cellular senescence similar to the senescent phenotype observed in replicative senescent cells, namely: increased cell size, multiple nuclei, flattened and granulated appearance, long protrusions, and irregular shape (Supplementary Figure S1b).

**Figure 1:**
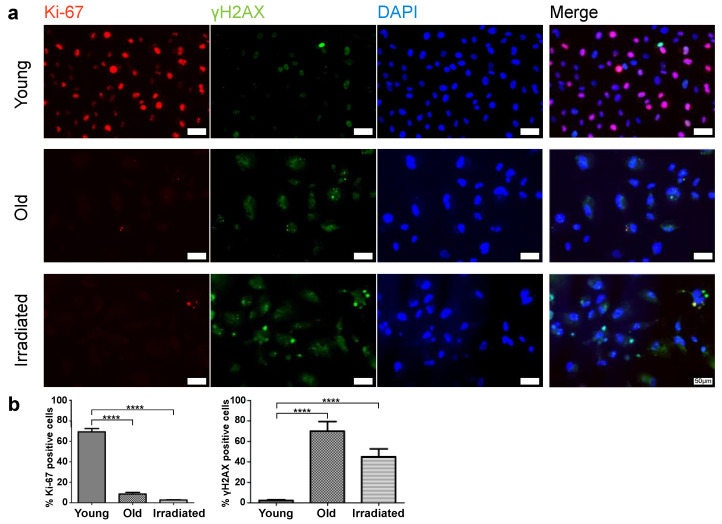
Assessment of Ki-67 and γH2AX expression in young and senescent HUVECs (old and irradiated). (**a**) Immunofluorescent co-detection of Ki-67 (red) and γH2AX (green); nuclear counterstain with DAPI scale bar 50 µm. (**b**) Quantification of the portion of Ki-67 positive cells and of cells showing the senescence-associated dotted pattern of γH2AX. Statistical analysis was made using unpaired *t*-tests. Significance levels are indicated as follows: **** *p* < 0.0001.

**Figure 2:**
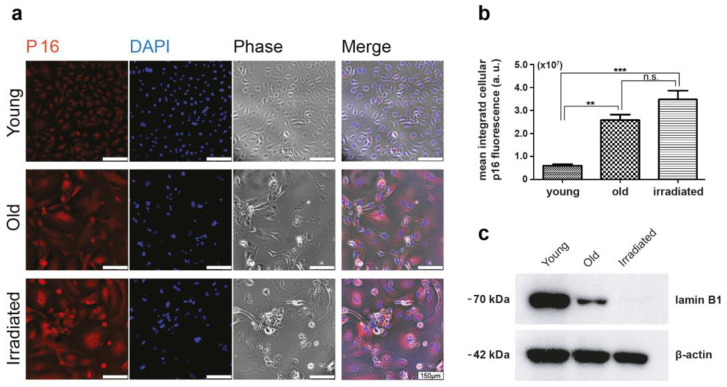
Assessment of p16 expression. (**a**) Immunofluorescent staining for p16 showing the different signal intensities of young versus senescent HUVECs (old and irradiated). P16 in red, nuclear staining in blue, and corresponding phase contrast images; scale bar 150 µm. (**b**) Differences in p16 expression were assessed as integrated cellular fluorescent signal intensities per field of view divided by the number of cell nuclei present (given in arbitrary units (a.u.)) by assessing at least five different fields of view using the Olympus software package cellSens and analyzed with unpaired *t*-tests using GraphPad Prism. Significance levels are indicated as follows: ** *p* < 0.01, *** *p* < 0.005, ns = not significant. (**c**) Western blot analysis of lamin B1 expression in young, old, and irradiated HUVECs. The detection of β-actin was used as an internal standard.

We conducted an extensive analysis to validate the senescent status of both types of generated HUVECs. The quantitative assessment focused on two key senescence markers: Ki-67, for measuring the proportion of cells ceasing proliferation [31], and γH2AX, for evaluating the increase in lasting DNA damage [32] (Figure 1). In addition, we examined the upregulation of the cell cycle inhibitor p16 [33], a well-established senescence marker, using immunocytochemical staining (Figure 2). Moreover, given the importance of senescent cells’ ability to evade apoptosis [34], we used immunofluorescence to visualize the augmented expression of apoptosis inhibitors Bcl-2 and XIAP [35,36] (Supplementary Figure S2). The multitude of assessed senescence indicators/markers used allowed us to omit the execution of the SA-ß-gal assay, an assay, which can lead to false positive results, e.g., in stressed cells, as described [37,38] and as observed by us.

This analysis revealed, as expected, that the portion of cells expressing the proliferation marker Ki-67 was minimal in both types of senescent cultures (Figure 1). While in cultures of young proliferating HUVECs, Ki-67 levels remained high, the percentage of Ki-67-expressing cells in senescent cultures was only about 9% (only one-third of these cells had an intense and two-thirds rather faint remnant Ki-67 signal) in the case of replicative senescence and about 3% in cultures prematurely senescent after irradiation. The expression of the double-strand break marker γH2AX in a pattern of few bright spots per nucleus, characteristic for senescent cells, was observed commonly in both types of senescent cell cultures, while in cultures of young HUVECs, this pattern was rarely detected, and γH2AX expression was limited to mitotic cells, where γH2AX is involved in DNA replication and appears in a finely dotted pattern (Figure 1). In phase contrast, one can observe typical alterations in size, shape, and granularity. As expected, the cell cycle-inhibitor p16 was expressed in the majority of cells of both types of senescence models, while in young cells, p16 was expressed to a much lower degree (Figure 2a,b). Further, we found that both types of senescent HUVECs expressed higher levels of the anti-apoptotic proteins Bcl-2 and XIAP than young HUVECs (Supplementary Figure S2), consistent with the higher resistance to apoptosis reported for senescent cells [34], even though, in the case of endothelial cells, this anti-apoptotic aspect might not be as pronounced as in fibroblasts [39]. In addition, we also assessed the protein expression level of an integral part of the nuclear lamina, lamin B1, which is commonly reduced in senescent cells [40,41]. Western blot analysis revealed that compared with young HUVECs, lamin B1 expression was low in cells senescent with propagation and virtually absent in cells senescent with irradiation (Figure 2c).

### 3.2. Senescent-Rendered HUVECs Are More Susceptible to Simvastatin-Induced Cell Death

In our study, we aimed to identify compounds that can selectively eliminate senescent endothelial cells while sparing young and healthy proliferating cells. We used two types of senescent HUVECs in parallel: those that were rendered senescent with exhaustive propagation and those that were rendered senescent with γ-irradiation, as well as young HUVECs with intact proliferation competence. To measure the senolytic effect in conjunction with possible negative effects on the proliferation ability of young cells, we plated different HUVEC types at their appropriate density in 384-well plates. For this, senescent cells were seeded at a density more or less completely covering the provided culture surface. Young cells were seeded at a sub-confluent density, allowing them to proliferate over the full course of the four-day experiments. We then exposed the cells to the tested substances at various concentrations for four days. We monitored the cells with live imaging and daily cell counts.

Our preliminary experiments had identified simvastatin, a classic blood cholesterol-lowering agent from the statin family of HMG-CoA reductase inhibitors [42], as a promising substance with senolytic potential in HUVECs. To analyze the senolytic capacity of simvastatin, we applied the substance at various concentrations ranging from 0.11 μM to 10 μM every other day for a period of four days. 

**Figure 3:**
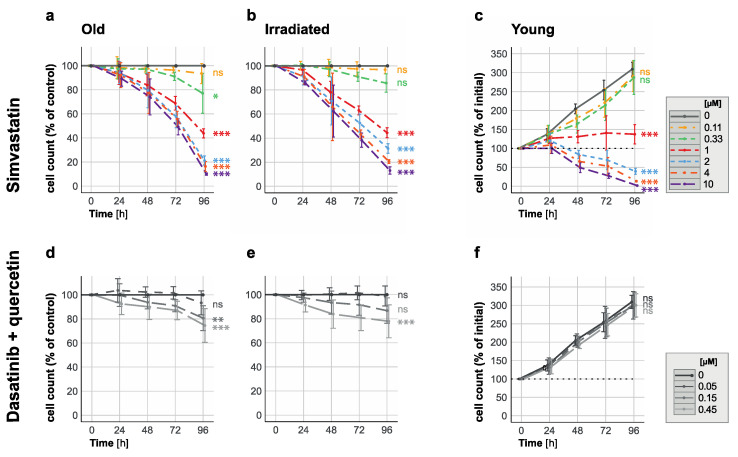
Elimination of senescent HUVECs and inhibition of proliferation in young HUVECs with 1 µM simvastatin. Time-response curves depict cell count changes for (**a**,**b**) senescent (replication/irradiation-induced) and (**c**) young HUVECs when exposed to varying simvastatin concentrations (ranging from 0.11 to 10 µM) over a 96-hour period. Additionally, time-response curves for (**d**,**e**) senescent and (**f**) young HUVECs treated with combinations of quercetin (10 µM) and dasatinib (0.05 µM, 0.15 µM or 0.45 µM) for 96 h are presented for comparison. Data in panels (**a**,**b**,**d**,**e**) are expressed as percentages of the untreated control (depicted as a solid gray line as 100%), while data in panels (**c**,**f**) are shown as percentages of the initial cell count (represented by the dotted line at 100%; solid gray lines represent the values for the untreated control). These results are based on three independent experiments conducted in duplicate. Statistical analysis was performed using ANOVA followed by a post hoc Dunnett’s Multiple Comparison test. Significance levels are indicated as follows: * *p* < 0.05, ** *p* < 0.01, *** *p* < 0.005, and ns = not significant.

We found that treatment with simvastatin at concentrations greater than 0.11 µM induced cell death in cultures of old HUVECs within four days in a concentration-dependent manner (Figure 3a). HUVECs that had become senescent upon irradiation died to a comparable or even greater extent (Figure 3b). Cultures of healthy young HUVECs treated with a concentration of 1 µM only were inhibited in their proliferation, while at concentrations of 2 µM and higher (Figure 3c), they also started to show signs of cell death (Figure 3c). As a positive control for senolytic activity, we used a previously published senolytic treatment [10] in which a combination of the kinase inhibitor dasatinib (at concentrations of 0.05 µM, 0.15 µM, or 0.45 µM) with the antioxidant quercetin (at 10 µM) was applied, as shown in Figure 3d–f. These concentration combinations of dasatinib and quercetin, as shown in our experiments, had an unexpectedly low eliminating effect on senescent cells, yet they completely spared young cells from cell death induction and proliferation hindrance at all tested concentrations.

In all experiments, we accounted for spontaneous cell loss in the senescent cultures, and therefore show the quantification of the treatment effect on the cell count in % of untreated control cells.

These in vitro results demonstrated the senolytic potential of simvastatin against both replication-induced and radiation-induced senescent HUVECs at concentrations above 0.11 µM and below 2 µM, prompting the question of the underlying mechanism and whether this potential is specific to simvastatin or common to all statins.

### 3.3. All Investigated Lipophilic Statins Showed Senolytic Activity, Which Could Be Negated with the Supplementation of Mevalonic Acid 

We aimed to determine whether the selective removal of senescent endothelial cells is a common property of statins, contingent on their blockage of the HMG-CoA reductase enzyme. To address this, we tested the senolytic capacity of several other statins and we investigated if such an effect would be negated with the concomitant provision of mevalonic acid—a molecular entity whose synthesis is directly blocked by statins [43] and which would therefore bypass the statin effect on the mevalonate/cholesterol synthesis pathway.

To address the first point, we tested the effects of simvastatin, two other lipophilic statins, i.e., lovastatin and atorvastatin, and the hydrophilic statin pravastatin [42]. As above, we exposed both types of senescent HUVECs as well as young proliferation-competent cells to these statins at concentrations ranging from 0.11 µM to 10 µM for a period of four days. In addition, we examined if the pre-activation of simvastatin might increase its senolytic activity, as some studies have reported that simvastatin would require activation with alkaline hydrolysis for its optimal activity in vitro [24].

**Figure 4:**
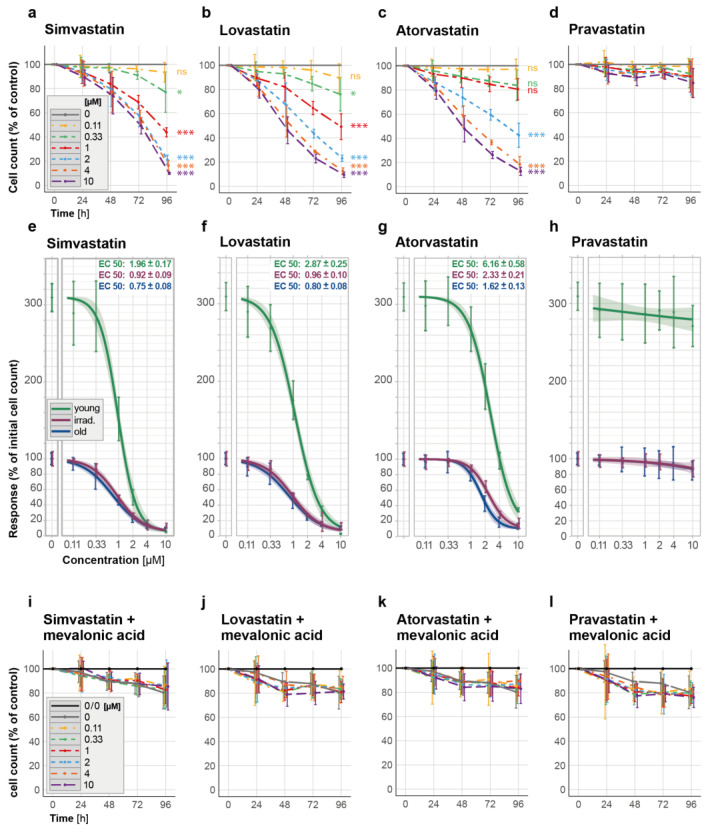
Elimination of propagation-induced senescent HUVECs (old) by statins and prevention thereof by mevalonic acid. (**a**–**d**) Time-response curves depict cell count changes for the effect of selected lipophilic and hydrophilic statins on the cell count of senescent HUVECs over a 96-h period: (**a**) simvastatin, (**b**) lovastatin, (**c**) atorvastatin, and (**d**) the hydrophilic pravastatin. (**e**–**h**) Concentration–response curves including EC50 values for old, irradiated, and young HUVECs show the cell counts (in % of the initial count) 96 h after exposure to different concentrations of statins. (**i**–**l**) Time–response curves for the cell count changes in old HUVECs exposed concomitantly to different concentrations of (**i**) simvastatin, (**j**) lovastatin, (**k**) atorvastatin, and (**l**) pravastatin and to 100 µM mevalonic acid. The used statin concentrations ranged from 0.11 to 10 µM and were applied for 96 h. Data in (**a**–**d**) are expressed as percentages of the untreated control (no statin, depicted as a solid gray line as 100%), in the graph legend for (**i**–**l**), 0/0 labels the untreated control (no statin and no mevalonate, black), while 0 labels the control only exposed to mevalonic acid but not to a statin (gray). These results are based on three independent experiments conducted in duplicates. Statistical analysis was performed using ANOVA followed by a post hoc Dunnett’s Multiple Comparison test. * *p* < 0.05, *** *p* < 0.005, and ns = not significant. All concentration–response curves, which were ascertained using two different lack-of-fit tests, are shown with 95% confidence intervals.

Our findings showed that lipophilic statins, including simvastatin, lovastatin, and atorvastatin, were effective in eliminating senescent HUVECs generated with exhaustive propagation while having little impact on young HUVECs (Figure 4a–c,e–g and Supplementary Figure S3a–c,i–k). Lovastatin required similar concentrations (starting at 0.33 µM, Figure 4b) for a comparable senolytic effect on old HUVECs, as previously observed for simvastatin (Figure 4a), whereas the concentrations required for a comparable senolytic effect were higher for atorvastatin, which started to eliminate senescent HUVECs only at concentrations above 1 µM (Figure 4a). Similar senolytic effects for all tested lipophilic statins were observed using irradiation-induced senescent cells (Supplementary Figure S3a–c). In contrast, in young HUVECs, the lipophilic statins became cytotoxic only at high concentrations (2 µM for simvastatin and lovastatin and over 4 µM for atorvastatin, Supplementary Figure S3i–k and Figure 4e–g) although a negative effect on the rate of proliferation was noted at a lower concentration already (of about 1 µM). 

The hydrophilic pravastatin, on the other hand, did not induce cell death in any type of senescent cells analyzed at the tested concentrations (Figure 4d and Supplementary Figure S3d), nor did it significantly affect young HUVECs (Supplementary Figure S3l and Figure 4h). The latter findings are consistent with the fact that hydrophilic statins, such as pravastatin [42], cannot penetrate the cell membrane on their own and thus require the presence of a suitable membrane transporter for cellular uptake, which is primarily expressed on liver and kidney cells [44].

As anticipated, the pre-activation of simvastatin resulted in the more effective removal of senescent HUVECs (Supplementary Figure S4a,b and Figure 3a,b), whereas in young HUVECs, activated simvastatin at the highest tested concentration of 1 µM did not cause a reduction in the cell count until day 4 (Supplementary Figure S4c).

After demonstrating that all the tested lipophilic statins have senolytic potential, we hypothesized that their ability to block the mevalonate pathway [42,45] is the molecular basis for their senolytic capacity. To test this hypothesis, we co-administered mevalonic acid (100 µM) during statin treatment, which provides the product of the step blocked here. Indeed, circumventing the blocked step of the mevalonate pathway suppressed the senolytic effect of all lipophilic statins studied (Figure 4i–k, presenting cell counts of old HUVECs, Supplementary Figure S3e–g, showing cell counts of irradiated HUVECs, and Supplementary Figure S3m–o, presenting cell counts of young HUVECs). Similar results were also observed when pre-activated simvastatin was used in combination with mevalonic acid (Supplementary Figure S4d–f). Expectedly, in the case of the hydrophilic pravastatin, adding mevalonic acid to the treatment did not result in any significant changes in senescent or young HUVECs (Figure 4l and Supplementary Figure S3h) nor in young HUVECs (Supplementary Figure S3p). 

These results demonstrate that the senolytic effects of lipophilic statins depend on the inhibition of the mevalonate pathway, as evidenced by the selective removal of senescent HUVECs by lipophilic statins, which can be blocked with the co-administration of mevalonic acid. 

### 3.4. Activated Simvastatin-Induced Apoptosis/Anoikis in Senescent HUVECs 

To study the underlying mechanism of how statins might act senolytically on HUVECs in more detail, we drew on the following two concepts. First, since senescent cells are viewed as damaged cells that have successfully evolved resistance to apoptosis, and the search for senolytic compounds often involves substances that would allow the induction of apoptosis again [9,46,47]. Second, prior studies have reported that statins, although applied at fairly high concentrations, can induce apoptosis in a variety of cancer cell lines and primary cells, including endothelial cells [48,49,50,51]. 

Based on this, we set out to ascertain whether apoptosis, and which specific subtype of apoptosis, plays a role in the elimination of senescent HUVECs when exposed to lower statin concentrations. A hallmark of the initiation of apoptosis is the activation of caspases, a family of cysteine proteases. Furthermore, the balance of pro-apoptotic (Bax) and anti-apoptotic (Bcl-2) members of the Bcl-2 protein family at the mitochondrial level regulates apoptosis [36]. Consequently, we explored whether statins differentially affect the pro- and anti-apoptotic balance in senescent versus young HUVECs.

Initially, we examined the expression levels of Bax and Bcl-2 because these proteins form mutually inhibiting heterodimers, and their quantitative ratio influences the decision of whether cells undergo apoptosis or not [36]. However, after exposing senescent or young cells to 0.33 µM activated simvastatin for 48 h, and we detected no significant change in the mRNA-level ratio of pro-apoptotic Bax and anti-apoptotic Bcl-2. 

**Figure 5:**
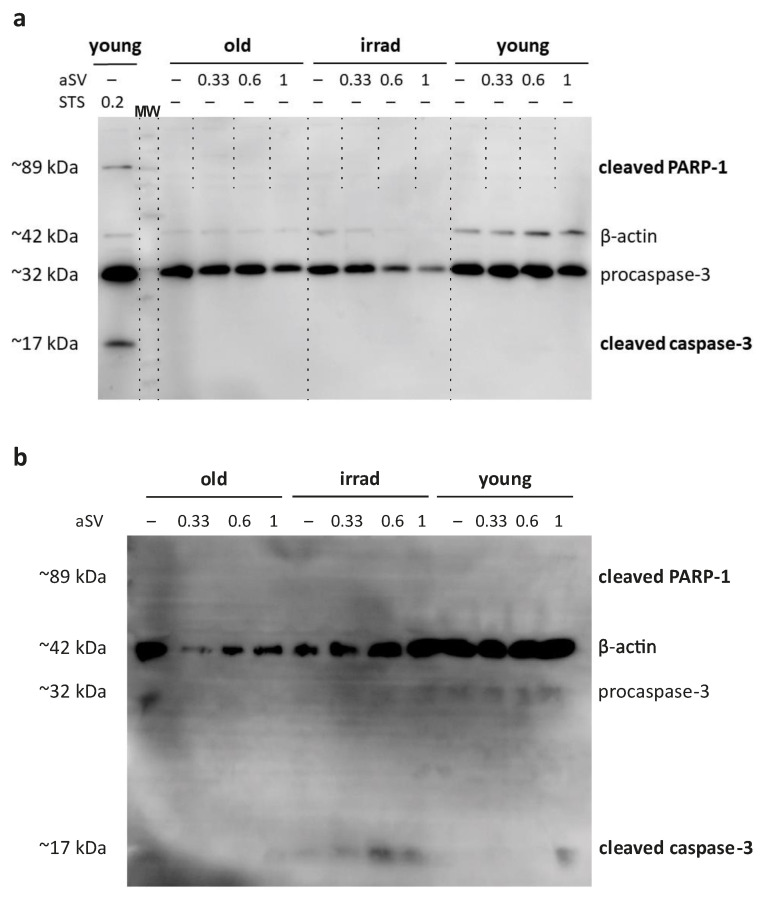
Western blot analysis of caspase-3 and PARP-1 activation. (**a**) Young, old, and irradiated HUVECs were exposed to 0.33 µM, 0.6 µM, and 1 µM activated simvastatin (aSV) for 72 h or alternatively, to 0.2 µM staurosporine (STS) for 3.5 h. (**b**) Caspase-3 and PARP-1 activation in supernatants collected after the initial 48 h of statin treatment. Samples in both cases, were analyzed with an antibody cocktail consisting of antibodies against pro-caspase-3, activated caspase-3, cleaved PARP-1, and actin as an internal standard.

To investigate the signs of apoptosis induction, we evaluated the proteolytic activation of key apoptosis mediators, i.e., caspase-3 and its downstream target PARP-1, in response to statins using Western blotting. Surprisingly, we did not observe evidence of cleaved, activated caspase-3 or proteolytically activated PARP-1 in adherent HUVECs exposed to 0.33 µM, 0.6 µM, or 1 µM activated simvastatin for 72 h (Figure 5a). In contrast, the application of staurosporine as a positive control for apoptosis induction did result in the proteolytic cleavage of caspase-3 and PARP1 in adherent HUVECs (Figure 5a), suggesting that simvastatin induces cell death with distinct characteristics.

To gain deeper insights into the nature of cell death induced by simvastatin, we used time-lapse microscopy. We closely tracked the morphological changes in and fate of HUVECs exposed to activated simvastatin, capturing images at a rate of 10 frames per hour. The resulting image sequences unveiled a distinct sequence of events: cells initially contracted, lost their contact with the 2D matrix, and progressed into a phase of pronounced membrane blebbing. Subsequently, observable cell movement came to an abrupt halt, signifying cell death. Notably, the characteristic feature of apoptosis, membrane blebbing, was exclusively observed in cells that had already detached from the matrix. This observed sequence of events led us to consider that the mode of cell death induced by statins might align with a specific subtype of apoptosis, known as anoikis [52,53]. Anoikis is mediated by the loss of cell–matrix contact and associated cell survival signals. To provide concrete evidence for our notion, we aimed to establish evidence for caspase activation in response to statins occurring only after cell deadhesion.

**Figure 6:**
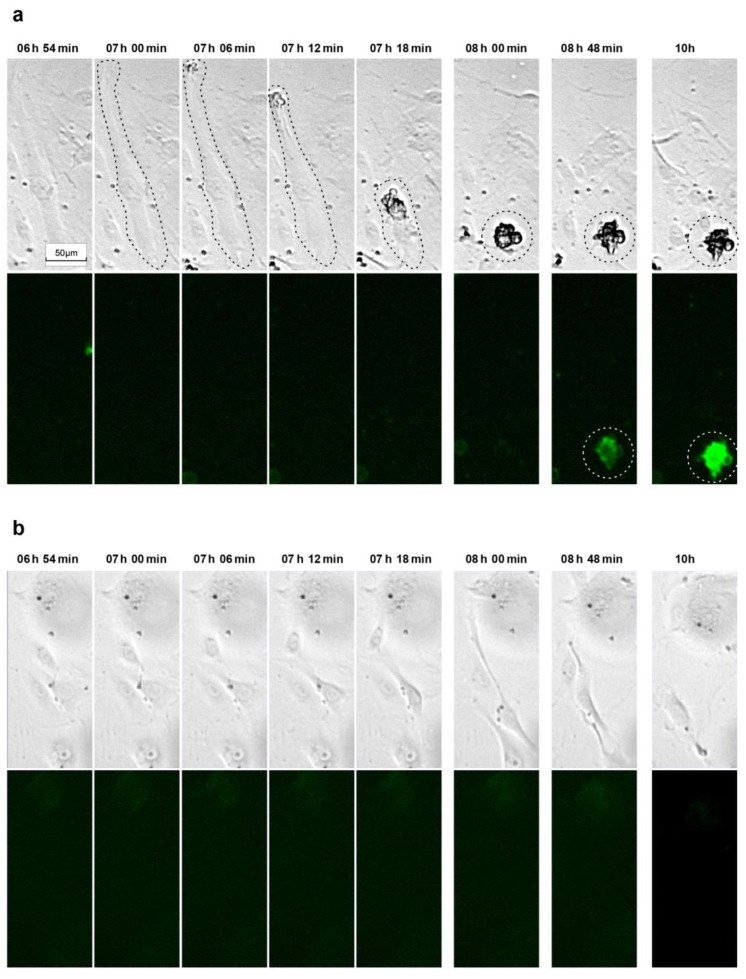
Examples of time-lapse sequences tracking morphological changes and caspase-3/7 activation in simvastatin-treated senescent HUVECs. (**a**) After exposing cells to a complete endothelial growth medium containing 0.33 µM activated simvastatin for 24 hours, the Caspase-3/7 Green Apoptosis Assay Reagent 4440 was added, and the cells were recorded at a frequency of 10 images per hour using both bright-field and fluorescence imaging (488/510 nm excitation/emission filter). The dashed lines highlight the cell of interest. The full-length video of the presented sequence is available in the Supplementary Material as Supplementary Movie S1. (**b**) For the control, vehicle-treated senescent cells at the same time points.

To this end, we used a fluorescence-based sensor for detecting caspase-3/7 activation to monitor the hypothesized induction of apoptosis in response to activated simvastatin over time. Starting at 24 h after the addition of 0.33 µM activated simvastatin, cells were recorded at the same frame rate as described previously, using both bright-field and fluorescence channels, to correlate the timing of caspase activation with morphologically discernible changes in the cells. Again, we observed that statin treatment resulted in cell detachment, followed by the occurrence of blebbing. Remarkably, caspase activation was only discernable in cells that had already severed their connection with the matrix (Figure 6, Supplementary Figure S5, and Supplementary Movies S1 and S2). This observed sequence of events, where caspase activation was only evident in cells that had already lost matrix contact, strongly supports the conclusion that the mode of cell death induced by statins is indeed anoikis. 

In an effort to further validate these findings and demonstrate the activation of caspase-3 in detached cells, we collected culture supernatants from senescent HUVECs exposed to activated simvastatin for 48 h. These supernatants were subjected to ultracentrifugation, and the pelleted proteins were subsequently analyzed using Western blotting with the same antibody cocktail used for adherent cells. The results revealed the presence of activated caspase-3 fragments in the cell culture supernatants of both types of senescent HUVECs (Figure 5b). Notably, samples from irradiated HUVECs exhibited more pronounced evidence of activated caspase-3 fragments compared with those from propagation-induced senescent HUVECs. While the observed signals were relatively low overall, it is essential to consider that the protein composition in the supernatants collected after the initial 48 h of incubation likely represents a snapshot of the protein content at the time of harvest rather than a cumulative picture, as a portion of the released proteins may have already degraded. 

Thus, our results provide support for the hypothesis that statins induce anoikis, a subtype of apoptosis induced by the loss of cell–matrix contact, in senescent HUVECs.

## 4. Discussion

Vascular dysfunction has been linked to many age-related diseases. Endothelial senescence, characterized by an irreversible growth arrest and the secretion of pro-inflammatory mediators that accompany senescence, is considered a culprit for vascular dysfunction. In the last decade, research focused on identifying senolytic compounds that allow for the selective elimination of such dysfunctional cells has led to the discovery of senolytic compounds that have been successfully tested not only in pre-clinical experimental animal models but also in a phase I pilot clinical study [54]. 

As senescent cells are known to be able to escape apoptosis, the search for senolytic compounds has in many cases focused on finding substances that would enable the induction of apoptosis in senescent cells [34,46,47]. Based on the knowledge that statins are compounds that can induce apoptosis in primary and tumor cells when applied at very high concentrations [48,49,50,51], we decided to explore the utility of statins for the selective elimination of senescent endothelial cells.

Here, we report that statins, widely used cholesterol-lowering drugs [17], had a senolytic effect on cultured vascular endothelial cells, as senescent HUVECs succumbed to statin exposure at lower concentrations than their proliferation-competent counterparts. Our decision to use actively proliferating young cells for control was driven by our intent to evaluate the potential side effects of the tested senolytic candidate substances on cell proliferation. Also, proliferating cells might exhibit higher susceptibility to toxicants compared with quiescent cells. Concurrently, we recognize the limitation that cells in a quiescent state may respond differently, potentially offering a more accurate reflection of in vivo conditions. The senolytic capacity that we initially observed on simvastatin at concentrations higher than 0.11 µM was not unique to simvastatin but was common to the other lipophilic statins studied, i.e., lovastatin and atorvastatin, while the hydrophilic pravastatin, unsurprisingly, and in accordance with its liver-specific uptake [44], did not show senolytic activity toward the exposed endothelial cells. 

In previous studies performed on non-senescent proliferation-competent endothelial cells, statins have been noted to induce apoptosis when applied at concentrations much higher than are clinically achieved during long-term therapy with these cholesterol-lowering drugs. For example, 3 to 30 µM of lovastatin caused apoptotic cell death in a majority of endothelial cells after 48 h [50], while in another study investigating apoptosis as early as 24 h, 2.5 µM simvastatin in HUVECs induced apoptosis at only a low level of about 10% [55]. The latter result is in accordance with our results, summed up in Figure 3a, assessing the effect of simvastatin treatment over the time course of 96 h. In rat pulmonary vein endothelial cells, the lipophilic statins simvastatin, lovastatin, atorvastatin, or fluvastatin applied at a very high concentration of up to 50 µM induced cell death within 24 h, which was associated with DNA fragmentation and activation of caspase-3 [51]. The hydrophilic pravastatin, which is taken up selectively in the liver and kidneys via a specific transporter [44], did not induce apoptosis in such cultured endothelial cells [51]. Our results, where the onset of toxic effects of lipophilic statins on proliferative primary endothelial cells occurred at concentrations greater than 1 µM for simvastatin and lovastatin and greater than 4 µM for atorvastatin, are consistent with these data. Overall, these studies demonstrated the ability of statins to induce cell death in proliferation-competent cells when used at high concentrations.

In our experiments, examining the differential response of senescent and non-senescent cells to statins, we demonstrated the senolytic potency of the lipophilic statins including simvastatin, lovastatin, and atorvastatin. We found that senescent HUVECs were eliminated by simvastatin and lovastatin at concentrations higher than 0.11 µM. In young cells, on the other hand, in accordance with the previous studies mentioned above, no visible induction of cell death was observed at these concentrations or up to 1 µM within the four-day treatment. Atorvastatin required higher concentrations for a comparable senolytic effect, and the senolytic concentrations started at 1 µM for both types of senescent HUVECs, whereas this compound was toxic only at concentrations of 4 µM and higher in proliferation-competent young cells. When comparing the senolytic effects of statins with the combination of dasatinib and quercetin that we used in our study as a reference [10], we found that dasatinib and quercetin eliminated senescent HUVECs at the used concentrations to a lower extent than statins. On the other hand, these compounds did not significantly impact the proliferation of young HUVECs.

The fact that all three investigated lipophilic statins were senolytic suggested that the underlying mechanism could be based on the inhibition of HMG-CoA reductase and thus the synthesis of mevalonic acid and the synthetic cascades dependent on it. Our results showing that the senolytic effect of statins could be blunted by the concomitant administration of mevalonic acid are in agreement with the observations of other research groups who showed that the apoptotic effect of statins on various tumor cells and endothelial cells was also prevented with the concomitant administration of mevalonic acid. Mevalonate, the synthesis of which can be directly inhibited by statins, is not only required for cholesterol production but is also important for the synthesis of sterol products such as geranylgeranyl pyrophosphate and farnesyl pyrophosphate; which deficiency is associated with statin-mediated apoptosis induction in endothelial cells [51,56]. It has been shown repeatedly that the prenylation of small G proteins such as RhoA, which are important for cell adhesion, is negatively influenced by statin administration. Exposure of cells to statins caused the retention of RhoA in the cytosol, reduced its activity at the cell membrane and impeded the formation of focal adhesion complexes, and compromised the integrity of the cytoskeleton, subsequently leading to apoptosis in endothelial cells [50,55] and other cell types [57]. Taken together, these findings suggest that statin-induced anoikis/apoptosis of senescent HUVECs too, is mediated by blocking mevalonate signaling. 

The question of the actual mode of statin-induced cell death of senescent HUVECs was addressed here. Using bright-field time-lapse video microscopy, we first showed that cells exposed to senolytic concentrations of simvastatin initially contract, then detach from the matrix, and subsequently show membrane blebbing, followed by apparent cell death. Such cell detachment is consistent with a previous report describing the detachment of healthy proliferative HUVECs in response to 1 or 5 µM simvastatin treatment [56]. Yet, although the authors of this study suggested that simvastatin fosters cell detachment by inhibiting prenylation and presumably caspase-8 activation, they acknowledged an open question: whether caspase activation precedes detachment or is a consequence thereof [56]. 

This question was addressed by our approach, where we visualized caspase activation in real-time using a fluorescent caspase-3/7 sensor. In these simvastatin-treated HUVEC cultures, we observed caspase activation only after cell detachment, supporting the notion that induction of cell death by statins might be primarily caused by anoikis, a form of apoptosis induced by the loss of cell–matrix contact [52,53]. This finding of caspase-3/7 activation in detached cells is in line with published Western blot data [56], where the active caspase fragment was found only in cell lysates derived from detached cells, not in those from adherent cells. In addition, this finding also fits the outcome of our Western blots where, similarly, we did not find activation of caspase-3 or its downstream target PARP-1 (Figure 5a) when analyzing lysates stemming from the adherent cells remaining after simvastatin treatment, whereas we did see hints on caspase-3 activation when analyzing protein extracts obtained from cell culture supernatants (Figure 5b). Overall, the presented literature data and our current results led us to conclude that lipophilic statins induce anoikis in senescent HUVECs.

Although the senolytic property of statins in endothelial cells has not been reported previously, several studies performed in pre-clinical models could indirectly support our results. For example, symptoms of experimentally induced pulmonary hypertension in rats were alleviated with simvastatin treatment [58]. In this case, the beneficial effects of statin treatment were coupled with the activation of caspase-3 in endothelial cells, as demonstrated in histological sections of pulmonary aortas [58]. Furthermore, the number of β-galactosidase positive, i.e., probably senescent cells, detected in the aortas of old rats exposed to oxidized low-density lipoprotein (OX-LDL), could be reduced with concomitant simvastatin treatment [59]. Nevertheless, it also has to be mentioned that long-term treatment of aged mice with simvastatin—in contrast with rapamycin treatment–did not increase their life span [60].

Our current study raises the question of whether short-term treatment with appropriate statin concentrations could provide a therapeutic benefit for “blood vessel rejuvenation” that surpasses the reported benefits of statins including lipid-lowering and anti-inflammatory properties as well as their ability to enhance NO synthesis [17,19,20]. Clinically, statins are often used as long-term therapy for patients at high risk of cardiovascular events, administered in daily doses of about 20 to 40 mg, resulting in maximum plasma concentrations between 0.01 and 0.1 µM [61,62]. The feasibility of transient therapy with short-term or intermittent high-dose statins is suggested by studies on oncological patients. For example, a daily dose of lovastatin of 25 mg/kg for 7 consecutive days was well-tolerated, with peak plasma levels reaching 2.32 ± 1.27 µM and mean plasma levels of 0.28 ± 0.09 µM [63]. More than one week of simvastatin treatment at a daily dose of 15 mg/kg has been described as being tolerated by oncological patients without severe side effects [64]. 

While the elimination of senescence endothelial cells is in general considered to beneficial [65], such an approach might also result in adverse effects. The elimination of p16^High^-expressing cells was detrimental to health and lifespan due the disruption of the blood–tissue barrier and resulted in liver fibrosis in experimental p16 knock-in mice [66]. The pitfalls of the elimination of senescence cells have been reviewed in more detail [3]. Thus, further thoroughly designed (pre)-clinical studies would have to be tackled in order to evaluate the possible benefits of “hit and run” treatment with high-dose lipophilic statins. 

The results reported here may invite the exploration of other naturally occurring HMG-CoA reductase inhibitors in addition to lovastatin [67] as lead compounds for senolytic therapeutics. For instance, policosanols, which are long-chain aliphatic alcohols found in sugar cane extracts, have shown lipid-lowering effects, possibly through increased AMP-kinase phosphorylation [68,69] or decreased HMG-CoA reductase expression [70]. Similarly, compounds like 4, 17(20)-pregnadiene-3, 16-dione (E- and Z-guggulsterone) from the resin of *Commiphora wightii* have demonstrated hypolipidemic activity [71] and appeared to directly inhibit HMG-CoA reductase in studies using HepG2 cells [72]. Such natural compounds might serve as candidates for developing novel senolytic therapies.

## 5. Conclusions

In summary, our study demonstrates the senolytic capacity of lipophilic statins—specifically simvastatin, lovastatin, and atorvastatin—on cultured human endothelial cells. Beyond a concentration threshold of 0.11 µM, these cholesterol-lowering drugs exhibit a distinct yet limited range of effectiveness in selectively eliminating senescent cells, offering promise in addressing age-related vascular dysfunction. The mechanism underlying statin-induced senolysis involves anoikis, triggered by the disruption of the mevalonate pathway. 

Our findings encourage the exploration of short-term, high-dose statin regimens as a means for vascular rejuvenation, introducing a novel dimension to the well-established benefits of these drugs. Additionally, our study suggests investigating alternative naturally occurring HGM-CoA reductase inhibitors for potential senolytic therapies. Thoroughly designed (pre)-clinical studies will be essential to evaluate the feasibility and risk associated with such interventive strategies.

Thus, this research not only advances our comprehension of senolytic interventions but also hints at a broader role for lipophilic statins in mitigating the impact of senescent endothelial cells. The translation of these findings into further investigations holds the potential to unveil new therapeutic avenues for these widely prescribed drugs.

## Data Availability

The data presented in this study are available upon request from the corresponding author.

## Supplementary Materials

The following supporting information can be downloaded at: https://www.mdpi.com/article/10.3390/cells12242836/s1, Supplementary Figure S1: Phase-contrast images documenting the development of senescence in HUVECs; Supplementary Figure S2: Immunofluorescent comparison of Bcl-2 and XIAP expression in young versus senescent HUVECs; Supplementary Figure S3: Effects of different statins on the cell count of irradiated and young HUVECs; Supplementary Figure S4: Effects of pre-activated simvastatin on the cell counts of old, irradiated, and young HUVECs; Supplementary Figure S5: Additional example of a time-lapse sequence tracking morphological changes and caspase-3/7 activation in simvastatin-treated senescent HUVECs; Supplementary Movies S1 and S2: Video sequences of senescent HUVECs exposed to activated simvastatin.
