# Supplementary material for: Lipophilic Statins Eliminate Senescent Endothelial Cells by inducing Anoikis-Related Cell Death
Source: Cells. 2023 Dec 14;12(24):2836. doi: 10.3390/cells12242836 (PMC10742095; doi:10.3390/cells12242836)
Supplement: Supplementary file 1 [file cells-12-02836-s001.zip › BB_ 2721158-Supplementary Material---.pdf]

Supplementary Material

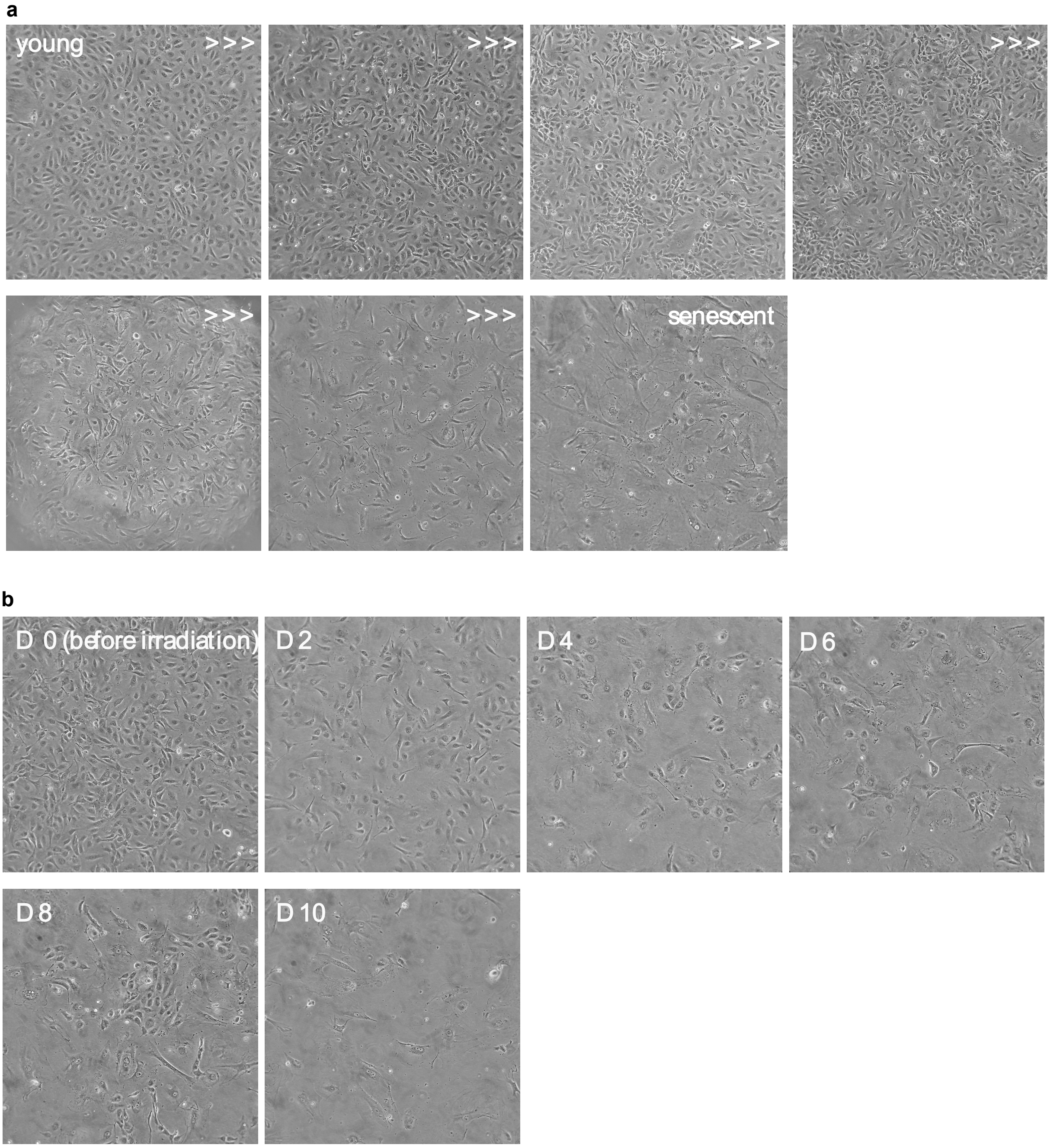

Supplementary Figure S1. Phase-contrast images documenting the development of senescence in HUVECs. (**a**) Propagation-induced replicative senescence: sequence of images of the changing morphology observed over time, showing alterations in size, shape and cell-cell contacts and increased occurrence of multi-nucleation. (**b**) Development of irradiation-induced premature senescence (single exposure to 10 Gy): images taken every other day over the course of 10 days (D 0 – D 10) are shown.

images were taken every five passages to enable the assessment of morphological changes in the cobblestone appearance typical of confluent HUVEC cultures, in cell size, in the extent of cell contacts and in the frequency of from passage 5 (P5) to passage 35.

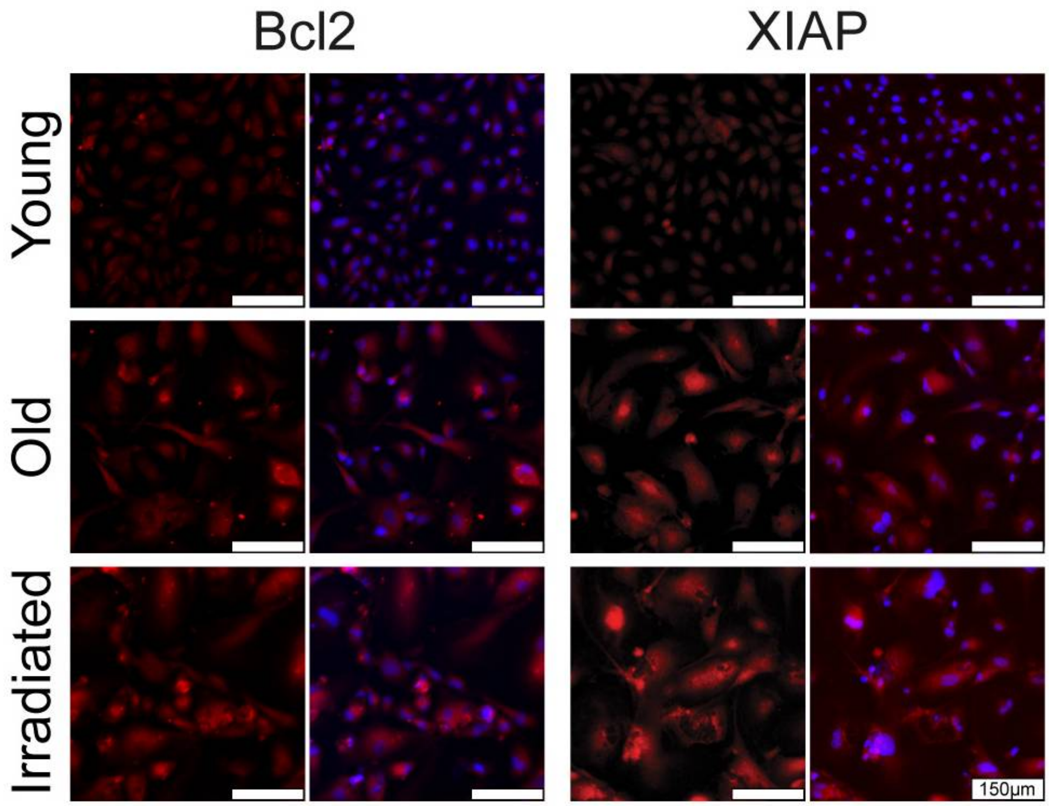

Supplementary Figure S2. Immunofluorescent comparison of Bcl-2 and XIAP expression in young versus senescent HUVECs. Cultures of young HUVECs and senescent HUVECs (old cells as well as irradiated cells) were stained using antibodies to Bcl-2 or to XIAP. Results are shown as separated red channel or as overlay with the nuclear stain. Bcl-2 and XIAP in red, nuclear staining in blue. Scale bar 150 µm.

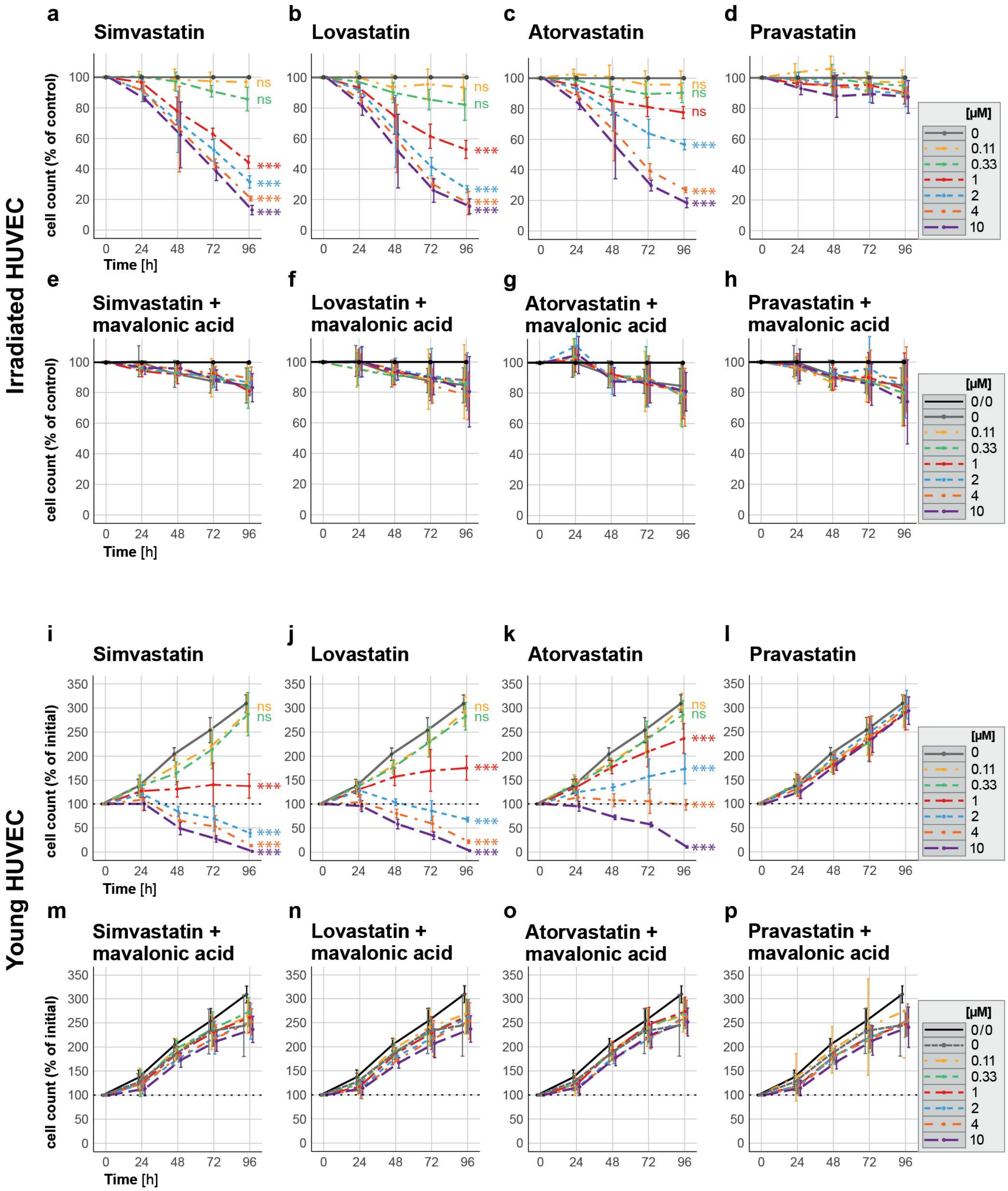

Supplementary Figure S3. Effects of different statins on the cell count of irradiated and young HUVECs and counteraction thereof by mevalonic acid. Statins have been applied at concentrations ranging from 0.11 to 10 µM for 96 h. (**a-c**) Effect of lipophilic statins and (**d**) of the hydrophilic pravastatin; (**e-h**) Effect of co-administration of 100 µM mevalonic acid during statin treatment; (**i-l**) Effect of various statins on proliferation and survival of young HUVECs; (**m-p**) Preventive effect of the simultaneous administration of 100 µM mevalonic acid.

Data in **a–d** are expressed as percentages of the untreated control (no statin, depicted as solid gray line as 100%). Data in **e-h** are expressed as percentages of the untreated control (containing neither a statin nor mevalonic acid; 0/0, solid black line at 100%). In these graphs, the condition without a statin but containing mevalonic acid is depicted as solid gray line.

Data in **i–p** are shown as percentages of the initial cell count. In the graphs **i-l** the solid gray line represents untreated control (no statin), while in **m-p** the untreated control represents the data for the control condition containing neither a statin nor mevalonic acid and is depicted as solid black line. These results are based on three independent experiments conducted in duplicates. Statistical analysis was performed using ANOVA followed by a post-hoc Dunnett's Multiple Comparison test. *$p<0.05$, **$p<0.01$, ***$p<0.005$, ns=not significant.

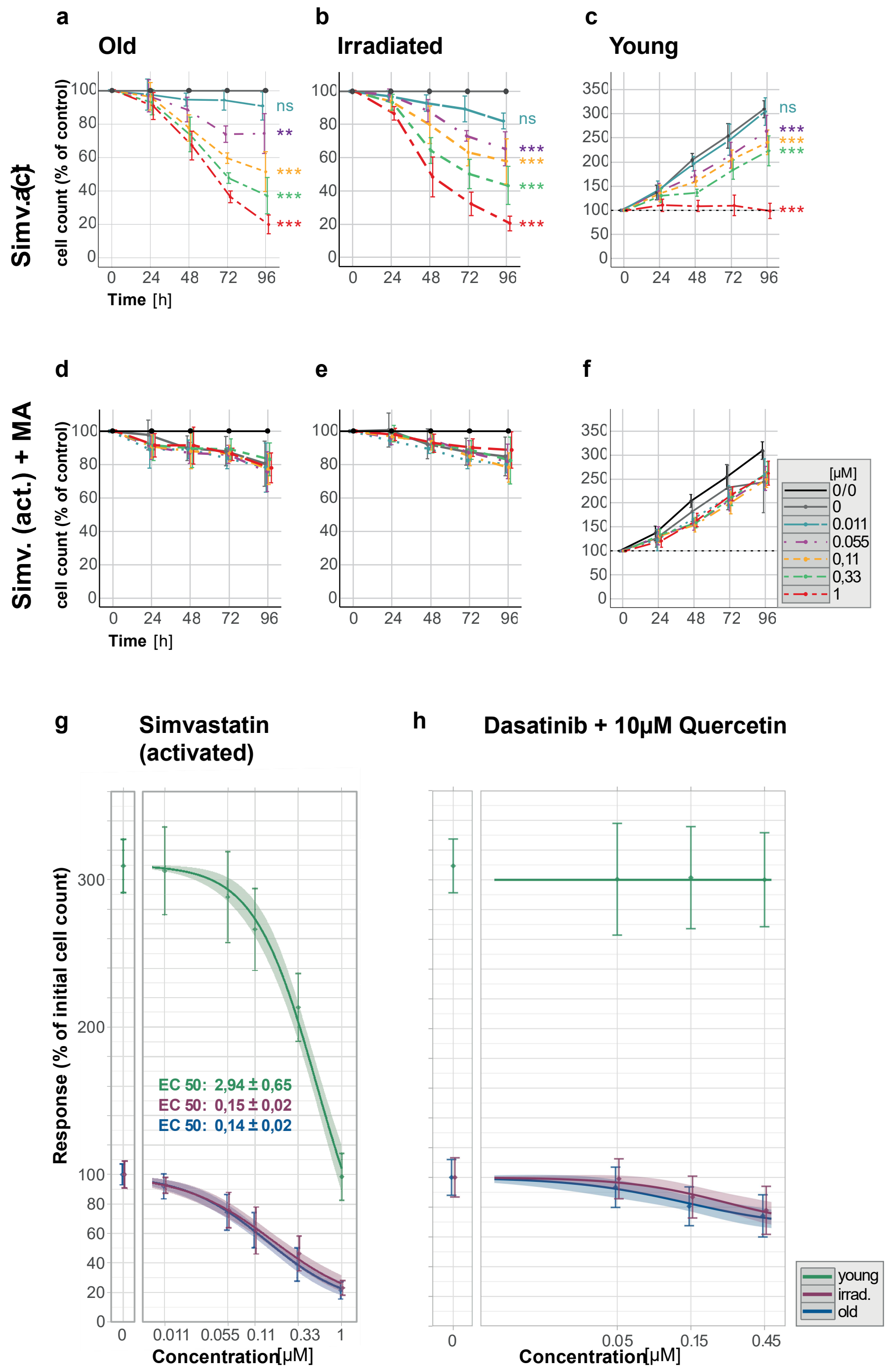

Supplementary Figure S4. Effects of pre-activated simvastatin on the cell counts of old, irradiated and young HUVECs. Activated simvastatin was applied at concentrations ranging from 0.011 to 1 μM for 96 hours. Change in cell counts of (**a-b**) senescent (replication/irradiation-induced) and (**c**) proliferating HUVECs exposed to the different concentrations of activated simvastatin alone or (**d-f**) in combination with 100 μM mevalonic acid (MA), respectively. (**g**) Concentration-response curves, including EC50 values, for old, irradiated and young cells show the impact of different concentrations of activated simvastatin on the resulting cell counts (in % of the initial count) at 96 hours. EC50 value of

young cells is an extrapolation of the slope of the curve, since the experimental conditions used did not result in a 50% reduction of the initial cell count. (**h**) For comparison, additionally concentration-response curves for old, irradiated and young cells treated with combinations of quercetin (10 µM) and dasatinib (0.05 µM, 0.15 µM or 0.45 µM) for 96 h are presented. Data in **a**, **b**, **d** and **e** are presented as percentage of the untreated control (depicted as solid gray line as 100%), while in **c** and **f** they are presented as percentage of the initial cell count. These results are based on three independent experiments conducted in duplicates. Statistical analysis was made using ANOVA followed by a post-hoc Dunnett's Multiple Comparison test. *$p<0.05$, **$p<0.01$, ***$p<0.005$, ns=not significant.

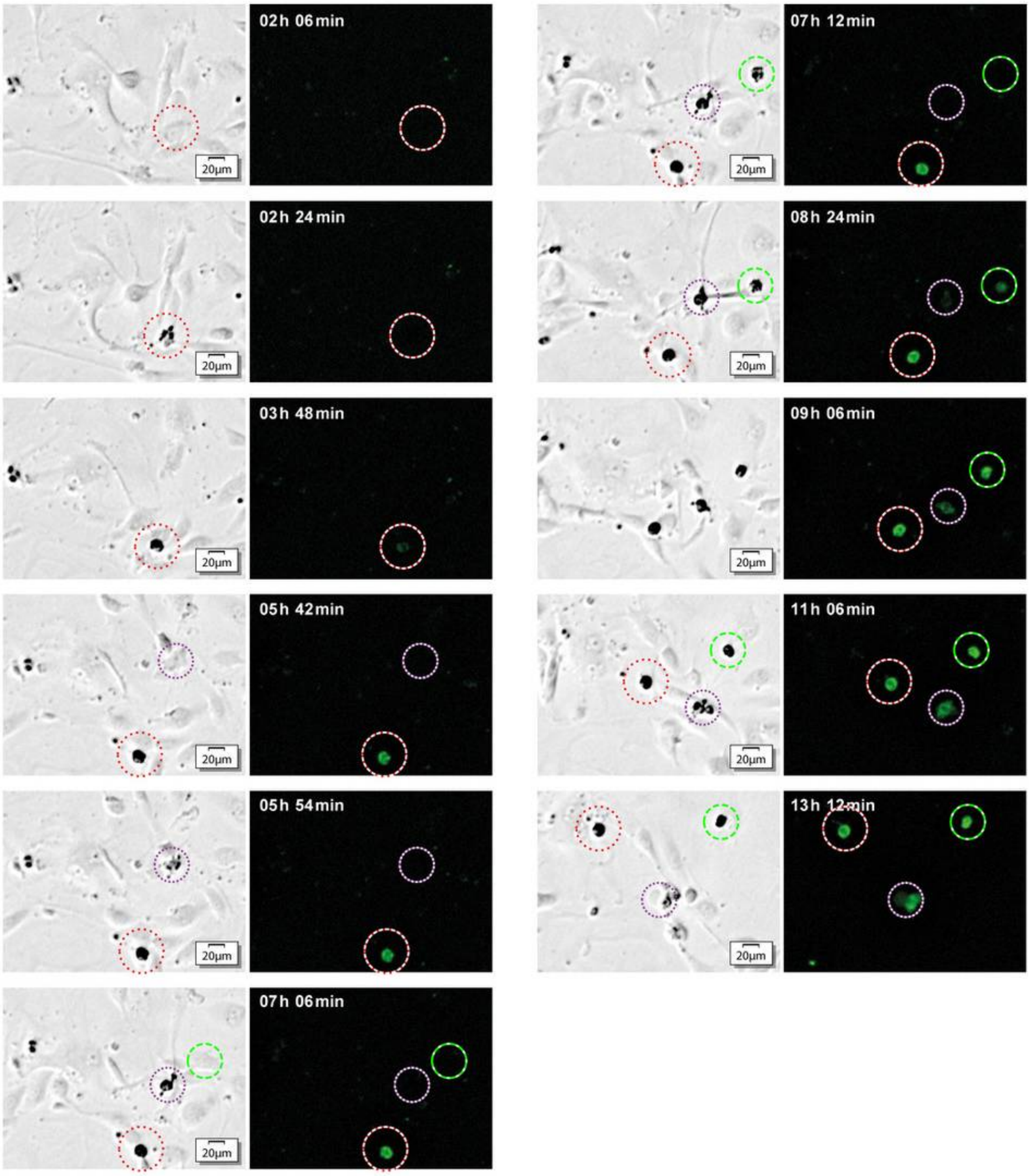

Supplementary Figure S5. Additional example of a time-lapse sequence tracking morphological changes and caspase-3/7 activation in simvastatin-treated senescent HUVECs. 24 h after exposing cells to complete endothelial growth medium containing 0.33 μM activated simvastatin, the Caspase-3/7 Green Apoptosis Assay Reagent 4440 had been added, and the cells were recorded at a frequency of 10 images per hour using both, bright-field and fluorescence (488/510 nm excitation/emission filter) imaging. Dashed circles highlight the three cells of interest and follow them through de-adhesion and caspase activation. The full-length video of the presented sequence is available in the Supplementary Material as Supplementary Movie S2.

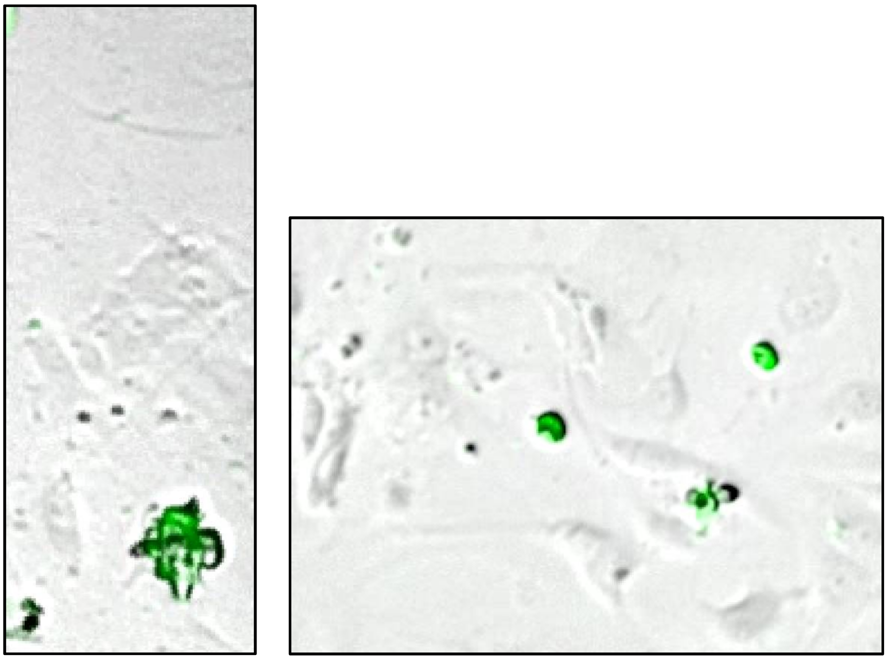

Supplementary Movies S1 and S2. Video sequences of senescent HUVECs exposed to activated simvastatin. These sequences of cells exposed to activated simvastatin at a concentration of 0.33 μM are compiled as overlay of the bright-field and fluorescence channel and are the source of the selected images shown in panels in **Figure 6** and **Supplementary Figure S5**.
